# Protective Effects of Irbesartan, an Angiotensin Receptor Blocker with PPARγ Agonistic Activity, against Estradiol Benzoate-Induced Endometrial Hyperplasia and Atypia in Female Rats via Modulation of TNFα/Survivin Pathway

**DOI:** 10.3390/ph14070649

**Published:** 2021-07-06

**Authors:** Mohamed A. Morsy, Wedad M. Abdelraheem, Maram El-Hussieny, Marwa M. M. Refaie

**Affiliations:** 1 Department of Pharmaceutical Sciences, College of Clinical Pharmacy, King Faisal University, Al-Ahsa 31982, Saudi Arabia; 2 Department of Pharmacology, Faculty of Medicine, Minia University, El-Minia 61511, Egypt; marwamonier@yahoo.com; 3 Department of Medical Microbiology and Immunology, Faculty of Medicine, Minia University, El-Minia 61511, Egypt; altaqwa.2012@yahoo.com; 4 Department of Pathology, Faculty of Medicine, Minia University, El-Minia 61511, Egypt; sama_feb.2009@yahoo.com

**Keywords:** endometrial hyperplasia, irbesartan, estradiol benzoate, tumor necrosis factor-alpha, survivin, cleaved caspase 3, interleukin-10, peroxisome proliferator-activated receptor gamma

## Abstract

Endometrial hyperplasia (EH) is a common gynecological problem and may progress to carcinoma. Early detection and management of EH are mandatory for the prevention of endometrial cancer. Activation of the renin–angiotensin system and angiotensin II signaling are involved in the progression of precancerous and cancerous lesions. However, no studies have evaluated the role of this system in estradiol benzoate (EB)-induced EH and atypia. Irbesartan (IRB), an angiotensin II receptor blocker with peroxisome proliferator-activated receptor gamma (PPARγ) agonistic activity was administered (30 mg/kg/d) in EB-treated (60 µg/100 g bodyweight, intramuscularly, three times per week) or untreated rats for 4 weeks. Uterine weight changes, malondialdehyde, superoxide dismutase (SOD), tumor necrosis factor-alpha (TNFα), survivin, cleaved caspase 3, interleukin-10 (IL10), and PPARγ were measured in addition to undergoing histopathological examination. Results showed that EB-induced EH and atypia significantly increased the uterine body weight, malondialdehyde, TNFα, and survivin, accompanied with significantly decreased SOD, cleaved caspase 3, IL10, and PPARγ, with typical histopathological changes of EH and atypia. Coadministration of IRB significantly prevented EB-induced biochemical and histopathological changes. The protective effects of IRB may be attributed to its anti-inflammatory and antioxidant properties, reduction of survivin, and increased levels of cleaved caspase 3.

## 1. Introduction

Endometrial hyperplasia (EH) is a significant gynecological problem, especially during childbearing age. It is a uterine pathology representing several morphological and endometrial changes. The hyperplastic changes originate from the uterine endometrial glands with an increase in the gland-to-stroma ratio compared with the regular endometrium. Unfortunately, about 40% of EH patients with atypia develop carcinoma, the most common fatal gynecological malignancy [1]. The revised 2014 WHO classification divides EH into two categories: (1) hyperplasia without atypia and (2) atypical hyperplasia, based upon the presence or absence of cytological atypia [2]. Hyperplasia without atypia is not associated with relevant genetic changes. However, about 1–3% of the cases progress to invasive cancer under the influence of prolonged estrogen exposure, especially if accompanied by relative progesterone insufficiency. Atypical EH exhibits mutations, which are typical for invasive endometrioid adenocarcinoma [2,3].

Suppression of apoptotic signaling pathways, including caspase 3 cascade, by increased estrogen levels, contributes to the development of EH and endometrial cancer (EC). Apoptotic cell death usually protects against DNA replication and repair errors, and somatic mutations. Thus, suppression of such protective mechanisms promotes EH, and subsequent malignancy [4]. The unopposed estrogen-mediated signaling in the endometrium increases inflammation via the release of the pro-inflammatory agents [5]. Moreover, induction of inflammation contributes to the initiation and progression of the disease via the release of interleukins (ILs), growth factors, and cytokines to facilitate immune cell recruitment and cell proliferation with sustained tumor growth [6]. One of the essential pro-inflammatory cytokines is tumor necrosis factor-alpha (TNFα) that induces the excessive formation of free radicals, rapid cell division, and DNA damage [4].

The imbalance between oxidative and antioxidant pathways is pivotal to the hyperplastic and cancerous changes in the endometrium. This imbalance starts and maintains an abnormal inflammatory state by activating pro-inflammatory cytokines such as TNFα. Subsequent activation and translocation of the transcription factor nuclear factor-kappa B (NF-κB) to the nucleus increases the expression of survivin, an inhibitor of the apoptotic process, upregulation of anti-apoptotic genes, excessive cell proliferation, differentiation, hyperplasia, and dysplasia [7,8]. Moreover, the inhibitor of apoptosis proteins (IAPs) can positively modulate NF-κB signaling to further support cell survival and tumorigenesis. Previous studies demonstrated the ability of c-IAP1 and c-IAP2 to interact with TNF receptor 1 and augment TNFα-stimulated NF-κB activation. Furthermore, the survivin–XIAP complex activates NF-κB, promoting further transcription of both growth and anti-apoptotic genes [9]. On the other hand, the anti-inflammatory cytokine interleukin-10 (IL10) opposes the TNFα/NF-κB/oxidative stress axis that is essential to endometrial hyperplasia and carcinogenesis. IL10 acts as an antitumor cytokine by inhibiting the NF-κB-induced pro-inflammatory cytokine expression. Furthermore, IL10 ameliorates the TNFα-induced reduction of superoxide dismutase (SOD) and increased lipid peroxidation [8,10].

Dysregulation of the endometrial renin–angiotensin system could predispose to EH and EC [11,12]. Previous studies implicated angiotensin II (AngII) and AngII type 1 receptor (AT1R) in the development of EC [13,14,15,16]. The ability of telmisartan, an AT1R blocker and a peroxisome proliferator-activated receptor gamma (PPARγ) agonist, to inhibit EC cell proliferation and tumor growth in nude mice adds further evidence to such hypotheses [17]. AngII-mediated activation of survivin signaling could explain its effect on tumor progression [18]. On the other hand, activation of PPARγ decreases the gene expression of survivin [19]. Survivin, which is widely expressed in different precancerous lesions and cancers, promotes cell survival and inhibits apoptosis [9]. Thus, drugs that could antagonize survivin hold promise for treating many forms of cancer [20,21]. Therefore, the aim of the present work was to investigate the probable protective mechanisms of the AT1R blocker with PPARγ agonistic activity irbesartan (IRB) against estradiol benzoate (EB)-induced EH and atypia in rats.

## 2. Results

### 2.1. Effect of IRB on Uterine Weight, Malondialdehyde (MDA), and SOD

MDA level and SOD activity were measured as markers of uterine oxidative stress. The EH-induced group showed a significant increase in uterine weights and MDA levels and a significant decrease in SOD activities compared to the normal control group. In contrast, the administration of IRB showed a significant decrease in uterine weights and MDA levels and a significant increase in SOD activities compared to the EH-induced group (Table 1).

### 2.2. Histopathological and Immunohistochemical Evaluation

#### 2.2.1. Macroscopic Examination

Uteri of control and IRB groups had no macroscopic abnormalities. There was marked dilation of the uterine horns in the EH group containing a thick turbid fluid. Uteri of the EH+IRB group had less uterine horn dilation than observed in the EH group and contained only serous fluid.

#### 2.2.2. Histopathological Evaluation

**Figure 1:**
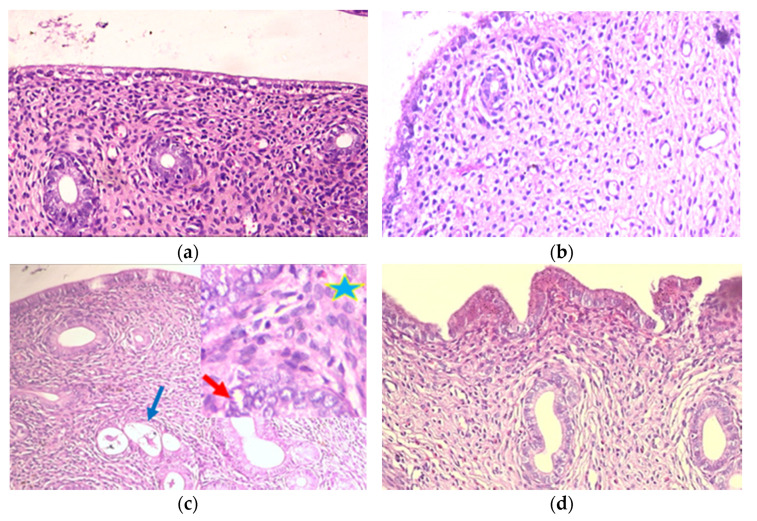
Effect of irbesartan (IRB) on histopathological changes in estradiol benzoate-induced endometrial hyperplasia (EH) and atypia in rats (hematoxylin and eosin, ×200; (*n* = 7/group). (**a**,**b**) Control and IRB groups, respectively, reveal no histopathological abnormalities. (**c**) In the EH group, the epithelial lining shows hypertrophy and stratification (blue arrow). The insert (×400) shows vesicular nuclei exhibiting atypical features with loss of polarity, anisonucleosis, and prominent nucleoli (red arrow), and the stomas show excessive leukocytic infiltration (asteroid). (**d**) The EH+IRB group shows a picture suggestive of almost normal-looking disordered endometrial glands, and the lining epithelium was low columnar.

Examination of control and IRB groups (Figure 1a,b; Table 2) revealed no histopathological abnormalities; surface and glandular epithelium were low cuboidal with fine eosinophilic cytoplasm and central regular nuclei. No glandular crowdedness with abundant endometrial stroma in between the glands. The EH group (Figure 1c; Table 2) showed EH features, as there were crowded irregularly shaped glands. The epithelial lining showed hypertrophy and stratification. Focal atypical EH was detected in variable areas. The irregularly shaped glands were compact together, with almost no stroma in between. Nuclei were vesicular, exhibiting atypical features with loss of polarity, anisonucleosis, and prominent nucleoli. The EH+IRB group (Figure 1d; Table 2) showed a picture suggestive of almost normal-looking disordered endometrial glands; few were cystic. The lining epithelium was low columnar with no features of EH.

#### 2.2.3. Evaluation of TNFα (Immunohistochemistry and ELISA)

**Figure 2:**
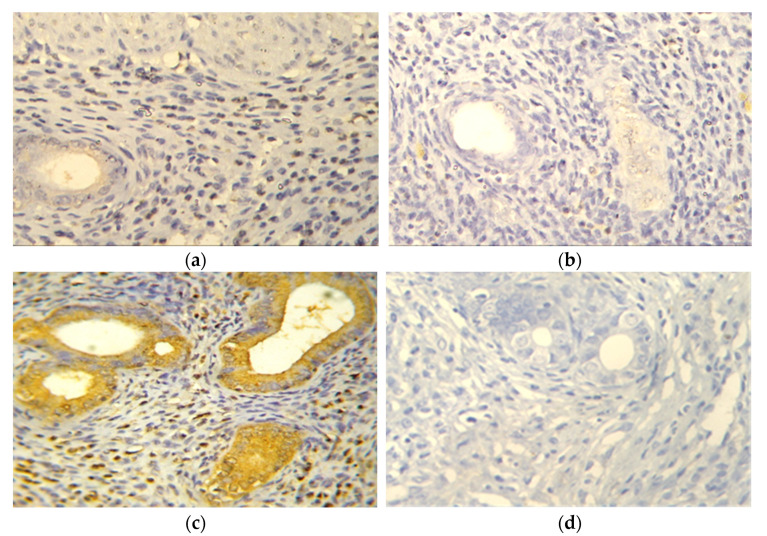

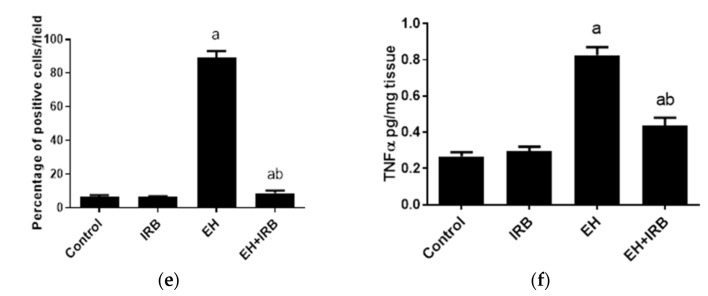
Effect of irbesartan (IRB) on uterine tumor necrosis factor-alpha (TNFα) in estradiol benzoate-induced endometrial hyperplasia (EH) and atypia in rats. Images are from representative sections of the rat uterus (×400) stained for detection of TNFα in (**a**) control, (**b**) IRB-treated, (**c**) EH, and (**d**) EH + IRB-treated groups. A semiquantitative analysis of TNFα expression (percent of TNFα positive cells/field) (**e**), as well as the protein levels of uterine TNFα level (pg/mg tissue) as determined by ELISA (**f**), are shown. Results represent the mean ± SEM (*n* = 6–7). ^a,b^ Significantly different (*p* < 0.05) from control and EH groups, respectively.

To evaluate the effect of IRB on the inflammatory pathway, the level of the uterine expression of the pro-inflammatory cytokine TNFα was evaluated. There was grade 1 (≤25% stained cells) TNFα expression in control, IRB, and EH+IRB groups (Figure 2a,b,d). On the other hand, the EH group showed grade 4 (>75% stained cells) TNFα expression (Figure 2c). Semiquantitative densitometrical analysis of uterine sections showed that TNFα expression was significantly higher in the EH group compared to the control group and significantly lower in the EH+IRB group compared to the EH group (Figure 2e). The same pattern was seen with the TNFα level measured with ELISA kit (Figure 2f).

#### 2.2.4. Evaluation of Survivin Expression

**Figure 3:**
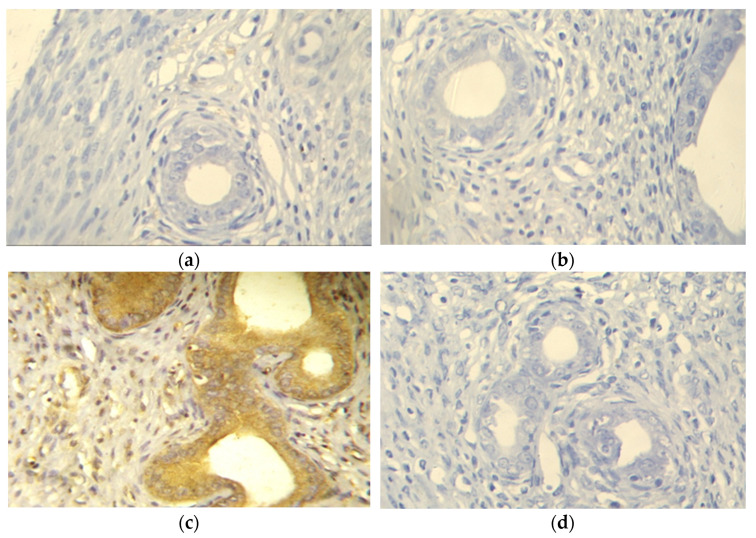

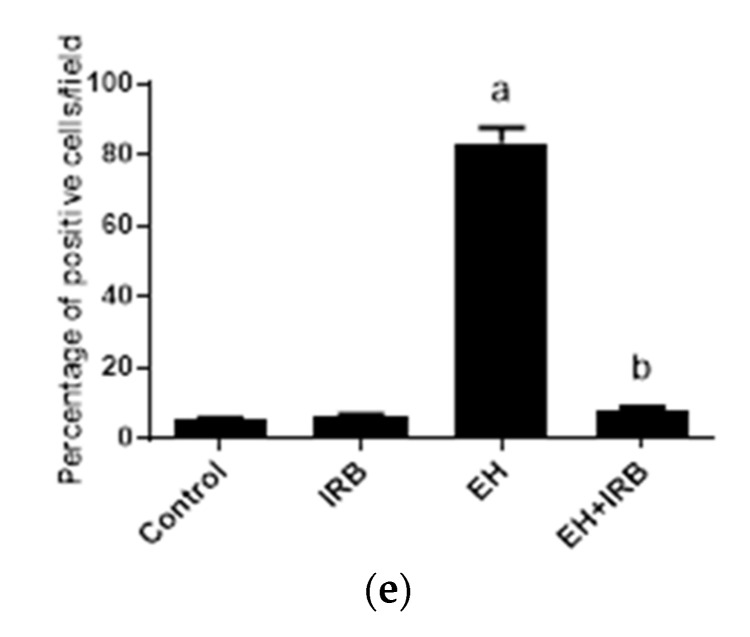
Effect of irbesartan (IRB) on uterine survivin immunohistochemical staining in estradiol benzoate-induced endometrial hyperplasia (EH) and atypia in rats. Images are representative sections of the rat uterus (×400) stained for detection of survivin in (**a**) control, (**b**) IRB-treated, (**c**) EH, and (**d**) EH+IRB-treated groups. Semiquantitative analysis of survivin expression in different groups was carried out (**e**). Results represent the mean ± SEM (*n* = 6–7) of the percentage of survivin positive cells/field. ^a,b^ Significantly different (*p* < 0.05) from control and EH groups, respectively.

To evaluate the effect of IRB on the cell-survival pathway, the level of the uterine expression of an inhibitor of apoptosis survivin was measured. Negative survivin expression was observed in control, IRB, and EH+IRB groups (Figure 3a,b,d). In contrast, the EH group showed positive survivin expression (Figure 3c). Semiquantitative densitometrical analysis showed that survivin expression was significantly higher in the EH group compared to the control group and significantly lower in the EH+IRB group compared to the EH group (Figure 3e).

#### 2.2.5. Evaluation of Cleaved Caspase 3 Expression

**Figure 4:**
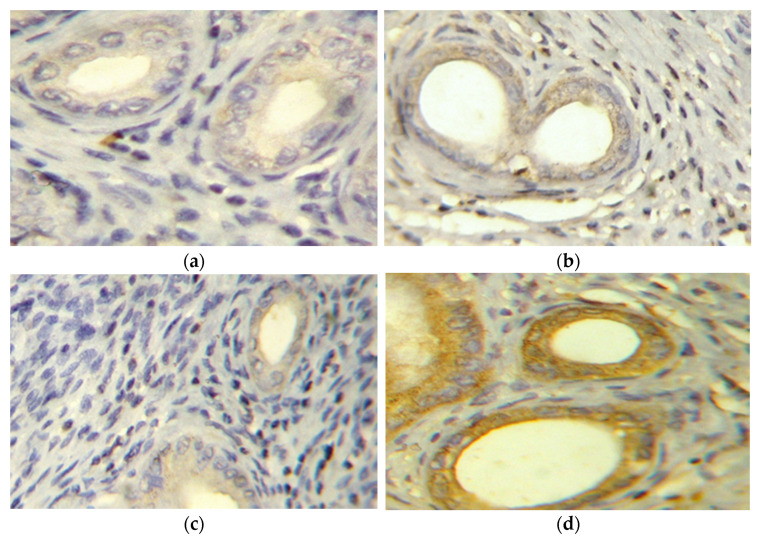

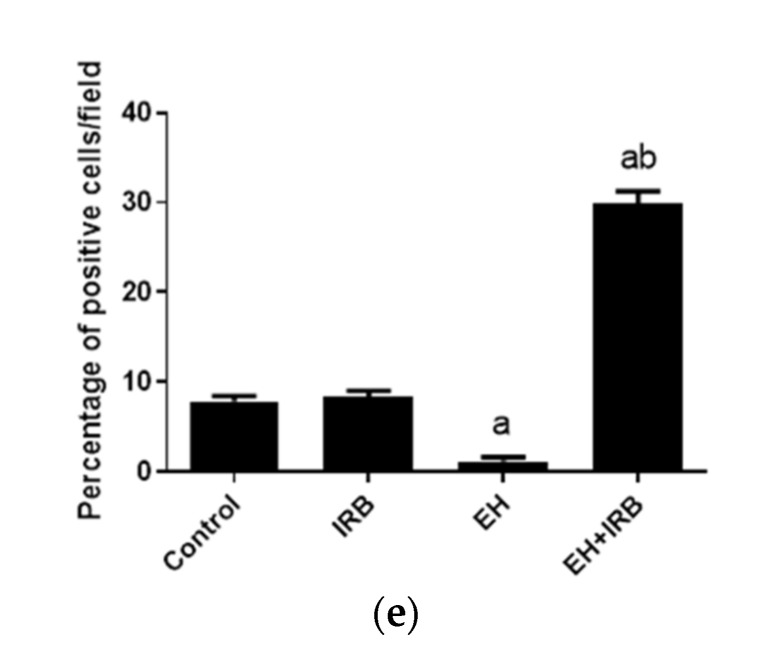
Effect of irbesartan (IRB) on uterine cleaved caspase 3 immunohistochemical staining in estradiol benzoate-induced endometrial hyperplasia (EH) and atypia in rats. Staining of cleaved caspase 3 in representative sections of rat uterus (×400) is shown in (**a**) control, (**b**) IRB-treated, (**c**) EH, and (**d**) EH+IRB-treated groups. Data in (**e**) show the results of the semiquantitative analysis of cleaved caspase 3 expression. Data represent the mean ± SEM (*n* = 6–7) of the percentage of cleaved caspase 3 positive cells/field. ^a,b^ Significantly different (*p* < 0.05) from control and EH groups, respectively.

Negative cleaved caspase 3 expression was observed in control, IRB, and EH groups (Figure 4a–c). Meanwhile, the EH+IRB group showed positive cleaved caspase 3 expression (Figure 4d). Semiquantitative densitometrical analysis showed that cleaved caspase 3 expression was significantly higher in the EH+IRB group compared to control and EH groups (Figure 4e).

### 2.3. Effect of IRB on IL10 Gene Expression

**Figure 5:**
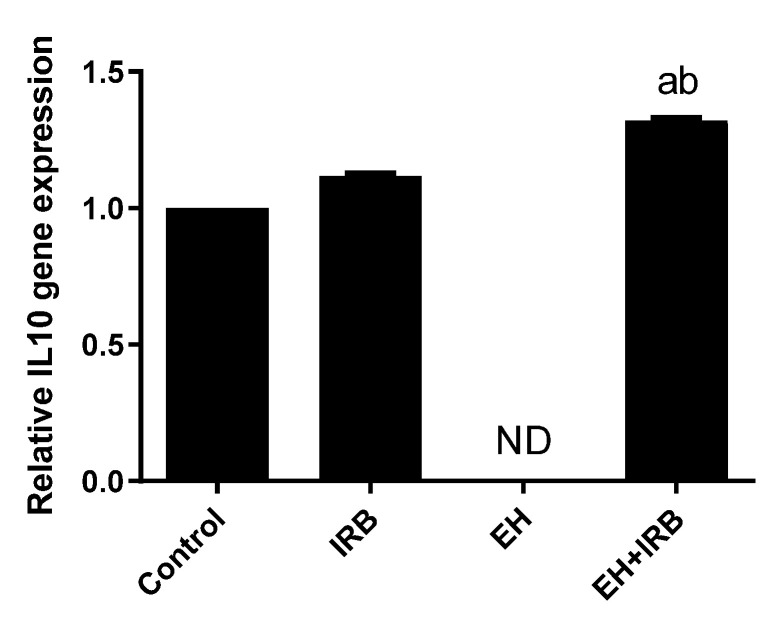
Uterine interleukin-10 (IL10) relative gene expression detected by real-time polymerase chain reaction. Values represent the mean ± SEM (*n* = 6). ^a,b^ Significantly different (*p* < 0.05) from control and EH groups, respectively. IRB: irbesartan; ND: non-detected.

Real-time polymerase chain reaction (PCR) was used to quantify the relative expression of the IL10 gene in different groups. Glyceraldehyde-3-phosphate dehydrogenase (GAPDH) was chosen as a reference gene to standardize mRNA expression. The analysis revealed the upregulation of the IL10 gene in the form of 1.1 fold in the IRB group and 1.3-fold in the prophylactic group (EH+IRB) compared with the control group. The IL10 gene expression in the EH group was not detected (Figure 5).

### 2.4. Effect of IRB on PPARγ Level

**Figure 6:**
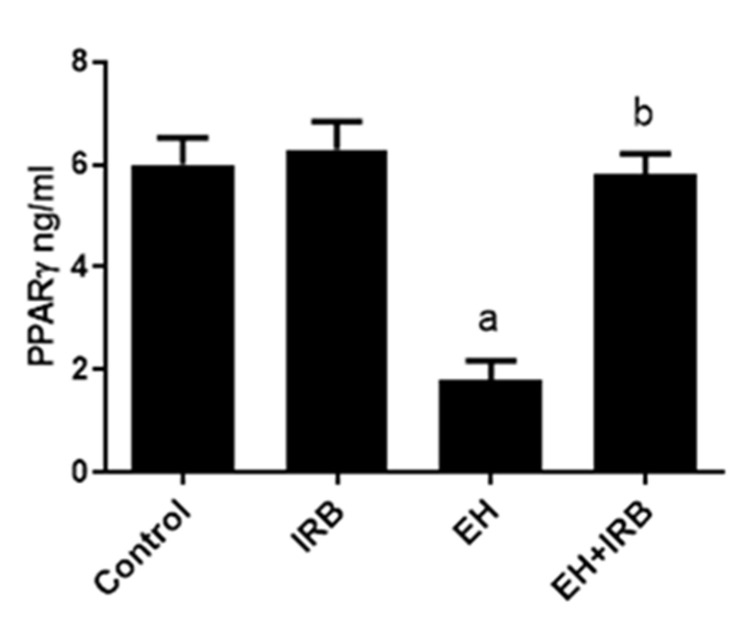
Effect of irbesartan (IRB) on peroxisome proliferator-activated receptor gamma (PPARγ) in estradiol benzoate-induced endometrial hyperplasia (EH) and atypia in rats. Values are mean ± SEM (*n* = 6–7). ^a,b^ Significantly different (*p* < 0.05) from control and EH groups, respectively.

The EH-induced group showed a significant decrease in uterine PPARγ levels compared to the normal control group. In contrast, the administration of IRB showed a significant increase in uterine PPARγ levels compared to the EH-induced group (Figure 6).

## 3. Discussion

Endometrial carcinoma is the most common gynecological malignancy, and EH is its precursor [1]. There is a strong relationship between the renin–angiotensin system and AngII receptors’ overexpression, and initiation and progression of EH and EC. Koyama et al. [17] evaluated the PPARγ agonistic effect of telmisartan, another AT1R blocker, as PPARγ induces apoptosis in uterine endometrial carcinoma [22]. This directed our attention to examine the possible unexplored protective mechanisms of IRB, an AT1R blocker with PPARγ agonistic activity [23], in EH. For example, IRB shows anti-inflammatory and antioxidant properties [24,25,26], two actions that are expected to be efficacious in the prevention of EH. The results showed that EB succeeded in the induction of EH as evidenced by the significant increase in uterine weights, MDA, TNFα, survivin with a concomitant decrease in SOD, cleaved caspase 3, IL10, and PPARγ, besides typical histopathological features of EH and atypia. Coadministration of IRB with EB significantly improved the biochemical and histopathological changes seen in the EB group.

Oxidative stress plays an important role in EH. Pejić et al. [27] reported that patients with EH and EC had elevated lipid peroxidation and decreased uterine SOD activities. Estrogen metabolites produce reactive oxygen species capable of inducing peroxidative damage to cellular membranes [28]. In agreement with the present study, previous reports [29,30] showed similar findings relating to the ability of EB to increase the lipid peroxidation product MDA, a known marker of oxidative stress, in EB-induced EH in female rats. Similarly, EB can increase oxidative stress in EH by decreasing the endogenous antioxidant enzyme SOD [29,30]. SOD effectively shields from oxidative stress by dismutation of superoxide radicals to hydrogen peroxide [31]. In the current study, IRB administration afforded protection to the uterus against EB-induced oxidative stress. Consistent with this result, several previous studies demonstrated similar findings relating to the ability of IRB to decrease oxidative stress through a significant reduction in MDA level with a significant increase in SOD activity [24,25], may be partly through AT1R blockade, as Ang II can result in oxidative stress [32], and PPARγ agonistic activity [33].

Inflammation plays an important role in the development and progression of EH [34]. In the present study, EB-induced EH was associated with increased inflammation, evident by increased uterine pro-inflammatory cytokine TNFα and decreased anti-inflammatory cytokine IL10. TNFα can contribute to the pathogenesis of EB-induced EH through induction of oxidative stress [35], generation of angiogenic factors [36], increase estrogen production [37], and activation of NF-κB-induced anti-apoptotic genes (Bcl-2 and survivin), inflammatory responses, and cyclooxygenase-2 [4]. In the present study, the increase in uterine TNFα is in harmony with the results of Abdelzaher et al. [38] who reported a significant increase in TNFα in estradiol valerate-induced EH in rats. In contrast, the expression of the IL10 gene did not change in patients with EH [39], although in the current study, EH was associated with decreased IL10 expression. The EH+IRB-treated rats showed lower uterine TNFα and enhanced IL10 compared to EH untreated rats. In agreement with these results, IRB decreased TNFα and increased IL10 in cyclophosphamide-induced ovarian damage in rats [40], high salt-induced hypertensive mice [41], and monocyte culture supernatants from hypertensive patients with left ventricular hypertrophy [26]. The anti-inflammatory effect of IRB may be in part due to AT1R blockade [16] and the PPARγ agonistic activity [42].

Survivin, a potent inhibitor of apoptosis, plays an essential role in EH [43,44]. He et al. [44] reported an increase in survivin in EB-induced EH, which is in line with the present study results. On the other hand, survivin was significantly lower in the EH+IRB group compared to the EH group. To our best knowledge, the present study is the first to report the effect of AT1R blockade on downregulation of survivin. However, as AngII is known to increase survivin [18,45,46,47,48], it is not a surprise it is inhibited by AT1R blocker. Moreover, PPARγ activation, which regulates cell proliferation and apoptosis, resulted in decreased survivin expression [19].

Consistent with the current study, previous reports [29,49] demonstrated similar findings in connection with the ability of EB to decrease cleaved caspase 3, an apoptosis marker, in EB-induced EH in rats. On the other hand, the EH+IRB-treated rats showed higher uterine cleaved caspase 3 compared to EH untreated rats. Compatible with these results, IRB increased cleaved caspase 3 in IRB-sensitive tumors in CBA mice [50]. The apoptotic effect of IRB may be partly owing to a decrease in survivin that inhibits the apoptotic process via suppressing caspase activities [9].

PPARγ has antiproliferative activity against EH [17] and EC [22]. In the present study, a significant decrease in PPARγ level was noticed in the EH group compared to the control group, while a significant increase in its level was detected in the EH+IRB group compared to the EH group. This reflects that PPARγ deficiency can be a contributing factor in mediating the pathogenesis of EH. As mentioned above, the PPARγ-mediated inhibition of EH can be due to a decrease in oxidative stress [33], inflammation [42], survivin [19], and an increase in cleaved caspase 3 [17]. In addition, PPARγ activation mediates IRB-induced adiponectin upregulation [51], which was found to be linked with decreased EC risk [52].

Finally, compatible with previous reports [29,30], the current study showed the characteristic histopathological changes of EB-induced EH and atypia. On the other hand, IRB was able to prevent the damage produced by EB administration, thus providing further support to the suggestive mechanism of action of IRB. Taken together, the protective effects of IRB against EB-induced EH and atypia may be mediated via anti-inflammatory (by modulating the pro-inflammatory cytokine TNFα and the anti-inflammatory cytokine IL10) and antioxidant (by modulating MDA and SOD) pathways, and possibly the reduction of survivin and increase in cleaved caspase 3 and PPARγ.

## 4. Materials and Methods

### 4.1. Chemicals

IRB was from Sanofi Egypt (Cairo, Egypt). EB was from Misr Pharma (Qaliubiya, Egypt). Polyclonal rabbit/anti-rat TNFα, survivin, and cleaved caspase 3 antibodies, biotinylated goat anti-rabbit secondary antibody (staining detection kit), TNFα ELISA kit, and quantitative real-time PCR kit were from Thermo Fisher Scientific (Waltham, MA, USA). PPARγ ELISA kit was from MyBioSource (San Diego, CA, USA).

### 4.2. Animals and Experimental Design

Adult female Wistar rats weighing 250–300 g were obtained from National Research Center (Giza, Egypt). Animals were kept in standard housing conditions in cages, 3 rats/cage, and left to acclimatize for one week. Rats were supplied with laboratory chow and tap water. This work was conducted in the Pharmacology Department, Faculty of Medicine, Minia University, Egypt, and the animal experimental protocol was approved (716:12/2020) by the Institutional Research Ethics Committee.

Rats were randomly assigned into 4 groups (*n* = 7 each). Group I received the vehicles (1% carboxymethylcellulose orally/day and intramuscular (i.m.) injection of olive oil 3 times/week) for 4 weeks. Group II received an oral daily dose of IRB (30 mg/kg) [53] and an i.m. injection of olive oil (3 times/week) for 4 weeks. Group III received 1% carboxymethylcellulose orally/day and i.m. injection of EB (60 µg/100 g; 3 times/week) for 4 weeks [44]. Group IV received an oral daily dose of IRB (30 mg/kg) plus i.m. injection of EB (60 µg/100 g; 3 times/week) for 4 weeks.

### 4.3. Preparation of Uterine Homogenate

At the end of the experiment, animals were weighed and euthanized. Each uterus was weighed, and part of the uterus was kept at −80 °C. Another part of the uterus was used to prepare tissue homogenate, for biochemical analysis, in 20% w/v in ice-cold phosphate buffer (0.01 M, pH 7.4). The homogenate was centrifuged at 4000 rpm for 15 min at 4 °C, and the supernatant was kept at −80 °C till used.

### 4.4. Biochemical Analysis

#### 4.4.1. Determination of Uterine MDA Level

Lipid peroxidation was assessed as thiobarbituric acid reacting substance and expressed as equivalents of MDA, using 1,1,3,3-tetramethoxypropane as a standard. The results were expressed as nmol/g tissue [54].

#### 4.4.2. Determination of Uterine SOD Activity

Briefly, uterine homogenates were mixed with Tris-HCl (pH 8.2) and pyrogallol (15 mM), and the absorbance of the sample was monitored against blank at 420 nm over a period of 3 min. The activity of SOD was expressed as unit/g tissue [55]. One unit of SOD could be defined as the amount of enzyme that inhibits the oxidation of pyrogallol by 50%.

### 4.5. Macroscopic Examination and Histopathological Evaluation

An examination of the uterus was performed to check for any macroscopic abnormalities. Regarding histopathological evaluation, the horn was dissected from each rat at the middle third, fixed in 10% formalin for 24 h, processed, and embedded in paraffin wax. Serial sections were prepared and stained with hematoxylin and eosin. The evaluation was conducted in a blind fashion using light microscopy.

Grading of histopathological changes was based on the following findings: glandular irregularity and crowdedness, epithelial lining hypertrophy and stratification, focal atypical cellular changes (loss of polarity, anisonucleosis, and prominent nucleoli), and stromal leukocytic infiltrate. The severity of the changes was graded semiquantitatively depending on the degree of the microscopic abnormalities as follow: ‘0’ for no changes, ‘+’ for mild changes, ‘++’ for moderate changes, and ‘+++’ for severe changes.

### 4.6. Immunohistochemistry

Paraffin-embedded sections on positively charged slides were used for staining. Briefly, uterine sections were deparaffinized in xylene and rehydrated in a graded alcohol series. Endogenous peroxidase was blocked with 0.3% hydrogen peroxide for 30 min to inactivate endogenous peroxides. Antigen retrieval was conducted by microwaving in sodium citrate buffer (pH 6.0). The sections were incubated with diluted primary antibodies TNFα (1:100, overnight), survivin (1:50, for 1 h), and cleaved caspase 3 (1:10, overnight). The sections were washed and then treated with biotinylated secondary antibody for 30 min at room temperature. Visualization was performed using 3,3′-diaminobenzidine chromogen, and Mayer’s hematoxylin was used for counterstaining. To evaluate the cytoplasmic TNFα expression, the percentage of positive cells was graded as follow: 0, no stained cells; 1, ≤25% stained cells; 2, >25% and ≤50% stained cells; 3, >50% and ≤75% stained cells; 4, >75% stained cells [56]. Cytoplasmic survivin and cleaved caspase 3 expression was evaluated as the percentage of positively stained cells and was considered positive when ≥10% of the cells showed cytoplasmic expression [57].

### 4.7. Real-Time PCR

Total RNA was extracted from homogenized uterine specimens using RiboZol RNA extraction reagent (AMRESCO, Solon, OH, USA) following the manufacturer’s instructions. cDNAs were synthesized using RevertAid™ First Strand cDNA Synthesis kit (Thermo Fisher Scientific). Real-time PCR was performed using Maxima SYBR Green qPCR Master Mix (Thermo Fisher Scientific) with specific primers in the Real-Time PCR Detection System (Kapa Biosystems, Wilmington, MA, USA). The sets of primers used were as follows: IL10 forward primer, 5′-AAAGCAAGGCAGTGGAGCAG-3′ and reverse primer, 5′-TCAAACTCATTCATGGCCTTGT-3′ [58] and glyceraldehyde-3-phosphate dehydrogenase (GAPDH) forward primer, 5′-GTCGGTGTGAACGGATTTG-3′ and reverse primer 5′-CTTGCCGTGGGTAGAGTCAT-3′ [59]. The SYBR green data were analyzed with a relative quantification to GAPDH as a reference gene. Fold changes of IL10 mRNA levels were calculated using the comparative cycle threshold method [60]. The fold change in gene expression was scaled relative to the control, where control samples were set at a value of 1.

### 4.8. ELISA

The inflammatory cytokine TNFα and the type II nuclear receptor PPARγ were determined in the uterine homogenate using TNFα and PPARγ ELISA kits according to the manufacturer’s instructions.

### 4.9. Statistical Analysis

Data were analyzed by one-way ANOVA followed by Dunnett Multiple Comparison Test. The values were represented as means ± SEM. Statistical analysis was conducted using GraphPad Prism software version 5 (San Diego, CA, USA). The differences were considered significant when the calculated *p* value was less than 0.05.

## Figures and Tables

**Table 1:** Effect of irbesartan (IRB) on uterine weights, malondialdehyde (MDA), and superoxide dismutase (SOD) in estradiol benzoate-induced endometrial hyperplasia (EH) and atypia in rats.

Group	Uterine Weight (g)	MDA (nmol/g Tissue)	SOD (U/g Tissue)
**Control**	1.0 ± 0.05	27.0 ± 2.7	3701 ± 374
**IRB**	0.9 ± 0.05	26.7 ± 2.4	4050 ± 275
**EH**	5.3 ± 0.20 ^a^	87.2 ± 5.2 ^a^	1440 ± 150 ^a^
**EH + IRB**	2.0 ± 0.06 ^ab^	33.7 ± 2.1 ^b^	2463 ± 238 ^ab^

At the end of the experiment (4 weeks), uterine weight changes, MDA, and SOD were determined. Values are mean ± SEM (*n* = 6–7). ^a,b^ Significantly different (*p* < 0.05) from control and EH groups, respectively.

**Table 2:** Effect of irbesartan (IRB) on the severity of histopathological lesions in estradiol benzoate-induced endometrial hyperplasia (EH) and atypia in rats.

Group	Glandular Irregularity and Crowdedness	Epithelial Hypertrophy and Stratification	Focal Atypical Cellular Changes	Leukocytic Cell Infiltration
**Control**	-	-	-	+
**IRB**	-	-	-	+
**EH**	+++	+++	+++	+++
**EH + IRB**	+	+	-	++

Score (-) is considered no change. Scores (+), (++), and (+++) are mild, moderate, and severe changes.

## Data Availability

Data are contained within the article or available upon reasonable request from the corresponding author.
